# MRP2 (ABCC2, cMOAT) expression in nuclear envelope of primary fallopian tube cancer cells is a new unfavorable prognostic factor

**DOI:** 10.1007/s00404-012-2589-7

**Published:** 2012-11-08

**Authors:** Agnieszka Halon, Verena Materna, Piotr Donizy, Rafal Matkowski, Jerzy Rabczynski, Hermann Lage, Pawel Surowiak

**Affiliations:** 1 Department of Pathomorphology and Oncological Cytology, Wroclaw Medical University, ul. Borowska 213, 50-556 Wroclaw, Poland; 2 Charité Campus Mitte, Institute of Pathology, Chariteplatz. 1 20/21, 10117 Berlin, Germany; 3 Department of Oncology and Division of Surgical Oncology, Wroclaw Medical University, pl. Hirszfelda 12, 53-413 Wroclaw, Poland; 4 Lower Silesian Oncology Centre, pl. Hirszfelda 12, 53-413 Wroclaw, Poland; 5 Department of Pathomorphology, Wroclaw Medical University, Marcinkowskiego 1, 50-368 Wroclaw, Poland; 6 Department of Histology and Embryology, Wroclaw Medical University, Chalubinskiego 6a, 50-356 Wroclaw, Poland

**Keywords:** MRP2, Primary fallopian tube carcinoma, Prognostic factor, Cisplatin resistance, Immunohistochemistry

## Abstract

**Objective:**

To determine the prognostic value of the immunohistochemical evaluation of the multidrug resistance-associated protein 2 (MRP2) expression, together with its subcellular localization in primary fallopian tube carcinomas (PFTCs).

**Methods:**

The immunohistochemical analysis was performed using samples originating from 70 patients with PFTCs.

**Results:**

(1) We documented that MRP2 can be localized in the plasma membrane (MRP2c), as well as in the nuclear envelope (MRP2n) of the PFTC cells. (2) Patients with more advanced stage, with progression of the disease and patients who died, showed significantly higher expression of the MRP2n. (3) Univariate and multivariate analyses showed that MRP2n is an unfavorable prognostic factor in PFTCs. (4) The analysis of the classic clinicopathological data revealed that only the FIGO stage had prognostic value, both in the univariate, as well as in multivariate analysis.

**Conclusions:**

(1) This study suggests that MRP2n is a new disadvantageous prognostic factor in PFTCs and (2) that expression in nuclear envelope can be associated with lower differentiation of cancer cells and their resistance to the cisplatin. (3) We have also confirmed independent prognostic value of FIGO stage in PFTCs.

## Introduction

Primary fallopian tube carcinomas (PFTCs) are uncommon tumors accounting for approximately 0.14–1.8 % of all gynecological malignancies. The annual incidence is 3.6 per 1 million women in the US. In Lower Silesia (the region in Poland of over 3 million inhabitants), PFTC is usually diagnosed and histologically confirmed at the frequency of 2–4 cases per year [1]. It is worth noting that the incidence of PFTCs appears to be increasing. Stewart et al. [2] demonstrated that the rate of fallopian tube cancer increased by 0.4 % annually from 1998 to 2003. Intriguingly, the rate of ovarian cancer decreased by 2.0 % per year [2, 3].

The similarities shared between fallopian tube carcinoma and epithelial ovarian carcinoma prompted to establish diagnostics criteria to distinguish fallopian tube carcinoma from other primary tumors (Hu et al. modified by Sedlis) [4–6] which stands as: (1) the tumor arises from the endosalpinx; (2) the histological pattern reproduces the epithelium of tubal mucosa; (3) transition from benign to malignant epithelium is found; (4) the ovaries are either normal or with smaller tumor than that in the tube.

However, the newest evidence indicates that ovarian cancers may mainly originate from the tubes: recent studies suggest that more than 50 % of high-grade serous carcinomas involving the ovary likely arise from fallopian tube epithelium [7, 8].

PFTC is associated with a very poor prognosis, especially in advanced stages of the disease [9–17]. This type of gynecological cancer has been described in the high-risk breast–ovarian cancer families with germ-line BRCA-1 and BRCA-2 mutations [18, 19]. Furthermore, molecular analysis revealed that unstable phenotype with highly scattered DNA ploidy patterns and p53 gene alterations are strongly connected with the development of PFTC [20].

Staging of the disease in accordance to the FIGO scale and the residual disease after initial surgery are the only, wide-accepted and reliable prognostic factors in PFTC [12, 13, 21–23]. The depth of tubal invasion in the cases limited to the fallopian tube [19, 23], and the presence of lymphocytic infiltration have been suggested to play an unfavorable prognostic role in PFTCs. The value of other commonly used prognostic factors did not find wider acceptance [12, 13]. They may be used as evidence to support prognosis, but none of them are independent prognostic factors.

The main function of multidrug resistance-associated protein 2 (MRP2) in cellular pathology is participation in the energy-dependent efflux pumps that reduce intracellular accumulation of anticancer agents [24]. In vitro experiments revealed that enhanced immunoreactivity of MRP2 could confer to cancer cell lines (including ovarian cancer) chemoresistance to platinum-containing anticancer drugs (cisplatin and carboplatin) and non-platinum-containing drugs, including methotrexate, vinblastine, and camptothecin derivatives [25–27].

In our previous study [27], we have reported that MRP2, one of the 48 human ABC-transporters, also called ABCC2 or the canalicular multiple organic anion transporter (cMOAT), may be present in the nuclear envelope of ovarian cancer cells and that such localization is typical for the cisplatin-resistant cancers.

MRP2 is localized in the apical membranes of canalicular cells in the liver [28], in the apical membranes of kidney proximal tubules, in epithelial cells of gall bladder, small intestine, colon, and lung [25]. We have also demonstrated that silencing of MRP2 expression is linked to increased sensitivity of tumor cells to cisplatin [29, 30]. We have shown that MRP2 is expressed in the nuclear envelope of stem cells in healthy human tissues [27]. This finding suggests that expression of MRP2 in the nuclear envelope may be typical not only for drug-resistant cells, but also for low differentiated cells.

This study aimed at immunohistochemical examination of the prognostic and predictive value of MRP2 expression and its subcellular localization in patients with PFTC.

## Patients and methods

### Patients

Immunohistochemical examination was performed retrospectively on tissue samples taken for routine diagnostic purposes. The study included all seventy patients with PFTC (Table 1) diagnosed or consulted in the Department of Pathomorphology, Wroclaw Medical University, Poland in the years 1982–2002. The cases were not stratified for known preoperative or pathological prognostic factors.

**Table 1:** Patient and tumor characteristics—survival analysis of the data (log-rank and *F* Cox tests)

Characteristics	No. (%)^a^	Log-rank *P* value	*F* Cox *P* value
All patients	70 (100)		
Age in years (mean 57.5)		0.1124	0.7593
≤50	16 (22)		
50–60	27 (39)		
>60	27 (39)		
Grade		0.7486	0.5880
1	13 (19)		
2	14 (20)		
3	14 (20)		
FIGO		**0.0143**	**0.0203**
IA	29 (41)		
IB	7 (10)		
IC	2 (3)		
IIA	11 (16)		
IIB	2 (3)		
IIC	1 (1)		
IIIA	6 (9)		
IIIB	7 (10)		
IIIC	3 (4)		
IV	2 (3)		
Histology		0.7236	0.4723
Endometrioid	26 (38)		
Undifferentiated	16 (22)		
Serous	15 (21)		
Transitional	8 (12)		
Clear cell	3 (4)		
Other	2 (3)		
Chemotherapy	14 (20)		

^a^Differences in the sum to 100 % in groups are due to rounding

Bold values indicate statistically significant (Hazard Ratio is 1.0684)

The study was approved by an Institutional Review Board (IRB). Tissue samples and paraffin blocks collected in our institution seem to be one of the largest collection worldwide, and the largest in Poland.

Age of patients ranged from 38 to 84 (mean 57.5). Histological classification of PFTC was performed according to the WHO ovarian tumor classification and the stage of disease was established based on the FIGO scale for fallopian tube cancer.

Histological classification revealed: 26 endometrioid cancers, 16 undifferentiated, 15 serous, 8 transitional, 3 clear cell and 2 another type. Thirty-eight patients were FIGO I stage, 14 FIGO II stage, 16 FIGO III and 2 FIGO IV (Table 1). The mean observation time was 52 months (range 2–178). Thirty-eight patients died with recurrence of the disease. Fourteen patients died without evidence of disease progression. The patients were monitored by periodic medical check-ups, CA-125 serum levels, ultrasonographic and radiological examinations. Progression of the disease was defined as clinical or biochemical recurrence of the disease.

Authors were not able to collect data concerning the residual disease after initial surgery in the investigated group of patients. Data concerning patients outcome, disease remission and overall survival time were collected based on hospital documentation and Lower Silesian Centre Registry database.

Tissue sampled from studied tumors were fixed in 10 % buffered formalin and embedded in paraffin. In each case, hematoxylin and eosin stained preparations were subjected to histopathological evaluation by two pathologists.

### Immunohistochemistry

Formalin-fixed paraffin embedded tissue was freshly cut (4 μm). Immunohistochemistry was done as described previously [27, 29, 30]. For the detection of MRP2, a monoclonal mouse antibody (clone M2I-4; Monosan, Uden, the Netherlands) was diluted 1:100 in the antibody diluent, background reducing (DakoCytomation, Poland). Tested sections were incubated with antibodies for 1 h at room temperature. Subsequent incubations involved biotinylated antibodies (15 min, room temperature) and streptavidin–biotinylated peroxidase complex (15 min, room temperature) (LSAB+, HRP, DakoCytomation, Poland). NovaRed (Vector Laboratories, UK) was used as a chromogen (10 min, at room temperature). All the sections were counterstained with Meyer’s hematoxylin. In each case, control reactions were included, in which specific antibody was substituted by the primary mouse negative control (DakoCytomation, Poland).

Control reactions included: positive control involving sections of human healthy liver, control reactions on tissue microarrays (Oligene GmbH, Berlin, Germany) with healthy human tissues, immunocytochemistry on the level of electron microscope, RT-PCR reactions, prediction of nuclear localization signal (NLS) in ABCC2 using the software “PredictNLS Online” (Version Jun 7, 2000) (http://cubic.bioc.columbia.edu/cgi/var/nair/resonline.pl). They were performed and described in detail previously [27, 29, 30].

### Scoring of immunostaining results

Intensity of the immunohistochemical reactions was appraised using the semi-quantitative immunoreactive score (IRS) scale [31], in which intensity of the reaction and percentage of positive cells were considered (Table 2). The final result represented a product of scores given for individual traits and ranged between 0 and 12. Intensity of the reactions was evaluated independently by two pathologists. In cases of divergences, the evaluation was repeated using double-headed microscope.

**Table 2:** Evaluation criteria of MRP2 expression using the immunoreactive score (IRS) [31]

Percentage of positive cells	Points	Intensity of reaction	Points
No positive cells	0	No reaction	0
<10 % of positive cells	1	Weak reaction	1
10–50 % of positive cells	2	Moderate reaction	2
51–80 % of positive cells	3	Intense reaction	3
>80 % of positive cells	4		

### Statistical analysis

Statistical analysis of the results took advantage of Statistica 98 PL software (Statsoft, Poland). The employed tests included ANOVA rank test of Kruskal–Wallis, Spearman’s rank correlation, Kaplan–Meier’s statistics and log-rank tests were performed using SPSS software (release 10.0; SPSS Inc., Chicago, IL, USA) to estimate significance of differences in survival times. We have also performed Kaplan–Meier’s statistics and log-rank tests on subgroup of 14 patients receiving cisplatin-based chemotherapy after the surgery. Using *F* Cox test, we have also performed multivariate survival analysis. Multivariate analysis covered data concerning age, tumor grade, FIGO stage, and MRP2 expression parameters.

## Results

### MRP2 immunostaining in PFTC

**Fig. 1:**
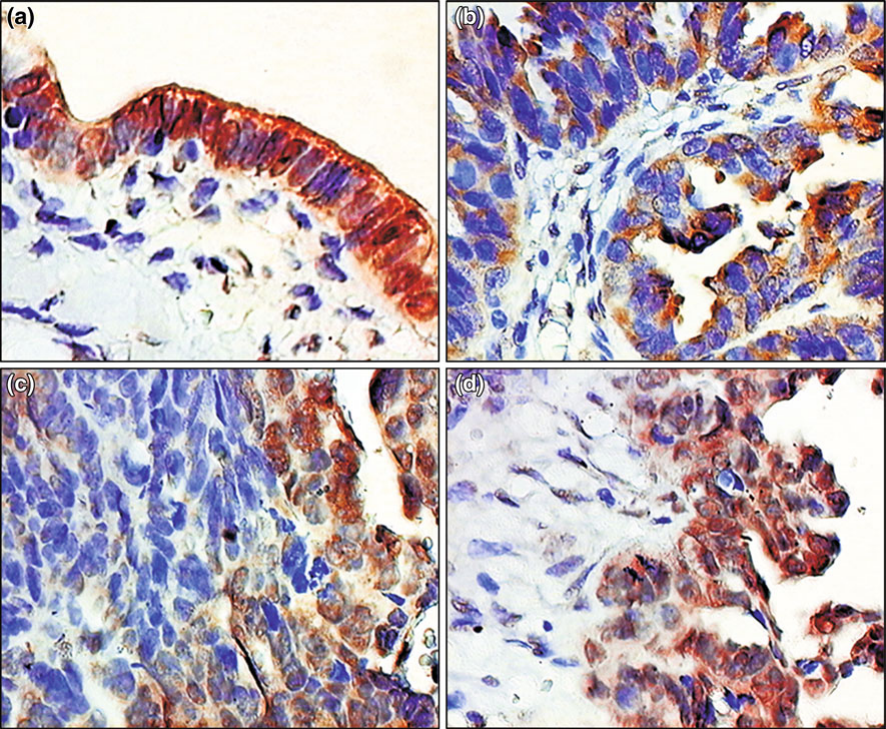
Immunohistochemical localization of MRP2 expression in: **a** normal fallopian tube epithelium, **b**–**d** primary fallopian tube carcinomas (hematoxylin, ×400)

We documented the expression of MRP2 in the normal ovarian epithelium in apical cell membrane of the majority of the cells and in the nuclear envelope in few cases (Fig. 1a). In case of PFTCs, the MRP2-specific staining reactions demonstrated a subcellular localization of MRP2 in the plasma membrane and cytoplasm (MRP2c) (Fig. 1b) as well as in the nuclear envelope (MRP2n) (Fig. 1c) and both localizations (Fig. 1d). The localization and the expression level of MRP2 were heterogenic in individual cases. Mean expression of MRP2c was 1.26 ± 1.77 SD (range 0–8) in the IRS scale and in case of MRP2n mean expression was 4.37 ± 3.65 SD (range 0–12) in the IRS scale. The expression of MRP2 in the nuclear envelope was significantly higher compared to its expression in the plasma membrane (*P* < 0.001).

### Relationships between expression of MRP2 and clinicopathological data

Patients with more advanced stage, and patients who died, showed significantly higher expression of the MRP2n (Table 3).

**Table 3:** Relationships between MRP2 expression and clinicopathological factors (ANOVA rank test of Kruskal–Wallis, Spearman’s rank correlation)

Studied parameter	MRP2 in the nuclear envelope	MRP2 in the plasma membrane
Age^a^	*P* = 0.8407	*P* = 0.5067
	*R* = 0.02444	*R* = −0.0807
FIGO	H (9, *N* = 70) = 18.14576	H (9, *N* = 70) = 11.51831
	*P* = 0.0319	*P* = 0.3303
Grade	H (9, *N* = 70) = 0.2527233	H (9, *N* = 70) = 1.140207
	*P* = 0.8813	*P* = 0.5655
Histology	H (9, *N* = 70) = 4.338052	H (9, *N* = 70) = 3.603356
	*P* = 0.3622	*P* = 0.4624
Progression of the disease	H (9, *N* = 70) = 7.075123	H (9, *N* = 70) = 0.6623821
	***P*** **=** **0.0078**	*P* = 0.6841
Death of the patient	H (9, *N* = 70) = 29.39694	H (9, *N* = 70) = 0.1655955
	***P*** **=** **0.00001**	*P* = 0.5993

^a^Spearman’s rank correlation

### Clinicopathological data and patients survival

The analysis of the classic clinicopathological data revealed that only the FIGO stage had prognostic value, both in the univariate, as well as in multivariate analysis (Table 1).

### MRP2 expression and patient survival

**Fig. 2:**
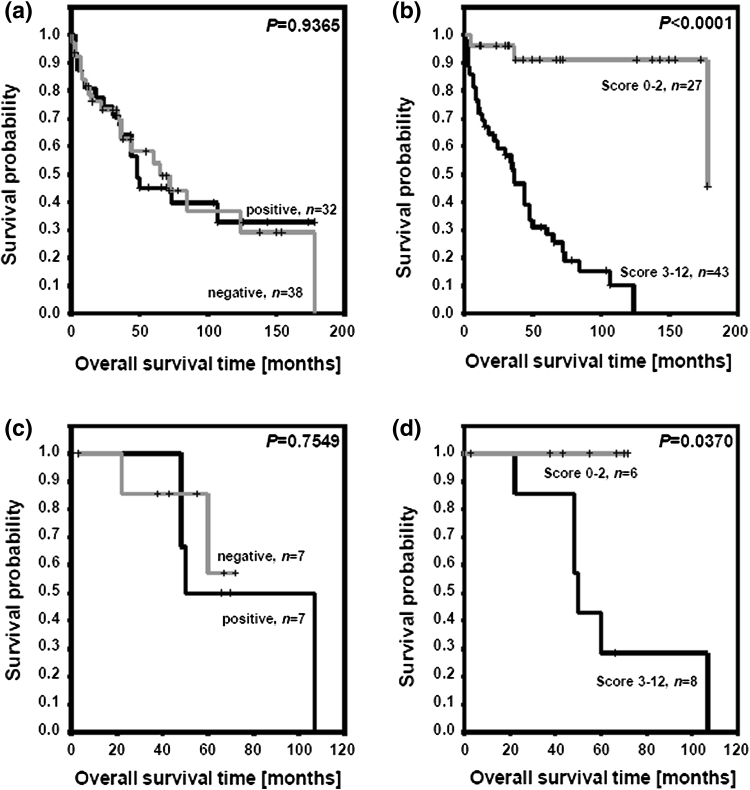
Kaplan–Meier curves for survival and expression of MRP2. **a** Cytoplasmic MRP2 expression and patients survival (entire studied group), **b** nuclear envelope MRP2 expression and patients survival (entire studied group), **c** cytoplasmic MRP2 expression and patients survival (chemotherapy-treated subgroup), **d** nuclear envelope MRP2 expression and patients survival (chemotherapy-treated subgroup)

Using the log-rank test and the Kaplan–Meier’s analysis, we revealed that the MRP2c expression has no prognostic value (Fig. 2a). In the case of the MRP2n, we observed shorter overall survival time in group of patients with higher expression of the MRP2n (IRS 3–12) compared to the group with lower expression of the MRP2n (IRS 0–2) (Fig. 2b).

Similarly, the analysis of the data obtained from 14 patients subjected to postoperative cisplatin-based chemotherapy revealed that the MRP2c (Fig. 2c) had no prognostic value, but patients with tumors showing higher expression of the MRP2n (IRS 3–12) had significantly shorter overall survival time comparing with the patient group with lower expression of the MRP2n (IRS 0–2) (Fig. 2d). Patients with expression of the MRP2n IRS 0–2 (*n* = 6) survived the entire observation period.

Multivariate analysis confirmed the lack of prognostic value of the MRP2c (*P* = 0.7665) and the significant role of the MRP2n (*P* = 0.0115).

## Discussion

In this analysis, we investigated two aspects: the significance of the MRP2 expression localized in the nuclear envelope (parameter described earlier [27] as disadvantageous prognostic and predictive factor during cisplatin therapy of the ovarian cancer) and the prognostic and predictive value of MRP2 expression in PFTCs. To the best of our knowledge, this is the first analysis of the MRP2 immunoreactivity in clinical specimens of PFTCs.

PFTC is relatively rare and is associated with poor prognosis [9–11]. From the various prognostic factors only the staging of the disease at the time of diagnosis in accordance with the FIGO classification has an established value [13, 21]. In this work, using univariate and multivariate analysis, we confirmed the significant prognostic value of the FIGO scale in the PFTCs. Simultaneously, we have not observed the prognostic value of the other clinicopathological parameters such as age, grade, and histological type. It is hard to establish the prognostic criteria for PFTC due to limited size of the group with the primary tubal cancer and specific histology of tumors. Primary fallopian tube malignant epithelial tumors do not reveal any specific histological structure. They are derived from Müllerian duct epithelium and indirectly from epithelium overlying the celoma. Thus, these neoplasms in their histology reflect the whole range of epithelial tumors commonly found in female genital tract beginning from uterine cervix finishing at ovaries [9].

Our results revealed that MRP2 can be localized both, in the plasma membrane, as well as in the nuclear envelope both in the normal ovarian epithelium and in the case of the PFTC. Considering our previous study [27] and data described here, we suggest that expression of the MRP2 in nuclear envelope in normal ovarian epithelium is characteristic for the stem cells of the epithelium. In the case of the PFTC cancer cells we have shown, similarly as in the case of the ovarian cancer [27], that expression of the MRP2 in the cytoplasmic membrane has no prognostic value, but its expression in the nuclear envelope can serve as an independent prognostic factor. In this work we documented for the first time that MRP2n is an independent unfavorable prognostic factor of the PFTC. We acknowledge the limitation of our study with its population size. However the study included all cases of fallopian tube cancer diagnosed or consulted in our Department in the years 1982–2002. In few older blocks (6 blocks from years 1992–1998), the evaluation of MRP2 intensity was more difficult but readable.

In above-mentioned studies, we also observed that in the group of the patients subjected to postoperative cisplatin-based chemotherapy, the high expression of MRP2n was the disadvantageous prognostic factor. In this group, 100 % of the patients with low (IRS 0–2) expression of the MRP2n survived the entire observation period. Unfortunately, in studied group only 14 patients were treated with chemotherapy after surgery, thus to determine the prediction value of the MRP2n expression in PFTC, the investigations of the bigger group of the patients is crucial.

Prognostic significance of the MRP2 expression was also widely discussed in other malignancies, such as esophageal squamous cell carcinoma [32], medullary thyroid carcinoma [33], pancreatic cancer [34], squamous cell carcinoma of the head and neck [35], lung cancer [36, 37], hepatocellular [38] and cholangiocellular carcinoma [39]. Although MRP2 confers the chemoresistance in several cancer types, its implication on gynecological neoplasms is still unclear [40–45]. MRP2 has been found to be overexpressed in several types of cisplatin-resistant cell lines [25] as a potential factor involved in ATP-dependent active efflux of the wide range of structurally unrelated cytotoxic agents. However, Ohishi et al. [42] demonstrated that the MRP2 mRNA level in serous papillary adenocarcinoma of the ovary was not associated with clinical outcome after platinum-based chemotherapy. Materna et al. [43] revealed a distinct tendency in correlation between high MRP2 mRNA expression and poor prognosis in ovarian carcinoma patients, but due to the low case number, the difference was statistically not significant. Immunohistochemical evaluation of the MRP2 expression was performed on 24 specimens of ovarian carcinoma, but this study also demonstrated no correlation with clinical response to platinum-based chemotherapy [44]. Interestingly, Ma et al. [45] using short hairpin RNA (shRNA) observed that the knock down of MRP2 effected in an increased intracellular cisplatin accumulation. Taking into account previous contradictory data, further studies are needed to fully determine the role of MRP2 in ovarian cancer progression.

In summary, in this work we have shown that MRP2 is localized in the nuclear envelope of the PFTC cells. This localization can be attributed to the lower differentiation of the cancer cells and their resistance to the cisplatin. We have also confirmed the significant prognostic value of the FIGO scale in PFTC.

This study supports the concept of MRP2 expression in nuclear envelope as possible marker of poor prognosis and resistance to cisplatin-based chemotherapy in PFTC patients but does not confirm its cytoplasmic expression value in those aspects. Estimation of MRP2n expression may provide valuable prognostic and predictive information proving that IHC may be a simple and accessible tool in identifying patients with worse prognosis and possible chemoresistance to cisplatin. Taking into account previous contradictory data and limitation of our study, further investigations are needed to fully determine the role of MRP2 in PFTC progression, prognosis and chemoresistance.

## References

[1] Reports based on data of the National Cancer Registry in Poland: http://85.128.14.124/krn/english/index.asp

[2] Stewart SL, Wike JM, Foster SL, Michaud F (2007). The incidence of primary fallopian tube cancer in the United States. Gynecol Oncol.

[3] Wethington SL, Herzog TJ, Seshan VE (2008). Improved survival for fallopian tube cancer: a comparison of clinical characteristics and outcome for primary fallopian tube and ovarian cancer. Cancer.

[4] Hu CY, Taymour ML, Hertig AT (1950). Primary carcinoma of the fallopian tube. Am J Obstet Gynaecol.

[5] Sedlis A (1978). Carcinoma of the fallopian tube. Surg Clin North Am.

[6] Ng P, Lawton F (1998). Fallopian tube carcinoma–a review. Ann Acad Med Singapore.

[7] Karst AM, Levanon K, Drapkin R (2011). Modeling high-grade serous ovarian carcinogenesis from the fallopian tube. Proc Natl Acad Sci USA.

[8] Levanon K, Crum C, Drapkin R (2008). New insights into the pathogenesis of serous ovarian cancer and its clinical impact. J Clin Oncol.

[9] Alvarado-Cabrero I, Young RH, Vamvakas EC (1999). Carcinoma of the fallopian tube: a clinicopathological study of 105 cases with observations on staging and prognostic factors. Gynecol Oncol.

[10] Baekelandt M, Nesbakken AJ, Kristensen GB (2000). Carcinoma of the fallopian tube. Clinicopathologic study of 151 patients treated at the Norwegian Radium Hospital. Cancer.

[11] Nordin AJ (1994). Primary carcinoma of the fallopian tube: a 20-year literature review. Obstet Gynaecol Surv.

[12] Hellström AC, Silfverswärd C, Nilsson B (1994). Carcinoma of the fallopian tube. A clinical and histopathologic review. The Radiumhemmet series. Int J Gynecol Cancer.

[13] Rosen AC, Ausch M, Hafner E (1998). A 15-year overview of management and prognosis in fallopian tube carcinoma. Austrian Cooperative Study Group for Fallopian Tube Carcinoma. Eur J Cancer.

[14] Rabczyński J, Ziółkowski P, Kochman A (1998). Primary fallopian tube carcinoma. Histopathology of 46 cases. Pol J Pathol.

[15] Papadimitriou CA, Markaki S, Lianos E (2009). Clinicopathological features of primary fallopian tube carcinoma: a single institution experience. Eur J Gynaecol Oncol.

[16] Pectasides D, Pectasides E, Papaxoinis G (2009). Primary fallopian tube carcinoma: results of a retrospective analysis of 64 patients. Gynecol Oncol.

[17] Liapis A, Bakalianou K, Mpotsa E (2008). Fallopian tube malignancies: a retrospective clinical pathological study of 17 cases. J Obstet Gynaecol.

[18] Rose PG, Shrigley R, Wiesner GL (2000). Germline BRCA2 mutation in patient with primary fallopian tube carcinoma: a case report. Gynecol Oncol.

[19] Aziz S, Kuperstein G, Rosen B (2001). A genetic epidemiological study of carcinoma of the fallopian tube. Gynecol Oncol.

[20] Pectasides D, Pectasides E, Economopoulos T (2006). Fallopian tube carcinoma: a review. Oncologist.

[21] Kosary C, Trimble EL (2002). Treatment and survival for women with fallopian tube carcinoma: a population-based study. Gynecol Oncol.

[22] Gadducci A, Landoni F, Sartori E (2001). Analysis of treatment failures and survival of patients with fallopian tube carcinoma: a cooperation task force (CTF) study. Gynecol Oncol.

[23] Peters WA, Andersen WA, Hopkins MP (1988). Prognostic features of carcinoma of the fallopian tube. Obstet Gynecol.

[24] Kruh GD, Belinsky MG (2003). The MRP family of drug efflux pumps. Oncogene.

[25] Taniguchi K, Wada M, Kohno K (1996). A human canalicular multispecific organic anion transporter (cMOAT) gene is overexpressed in cisplatin-resistant human cancer cell lines with decreased drug accumulation. Cancer Res.

[26] Liedert B, Materna V, Schadendorf D (2003). Overexpression of cMOAT (MRP2/ABCC2) is associated with decreased formation of platinum-DNA adducts and decreased G2-arrest in melanoma cells resistant to cisplatin. J Invest Dermatol.

[27] Surowiak P, Materna V, Kaplenko I (2006). ABCC2 (MRP2, cMOAT) can be localized in the nuclear membrane of ovarian carcinomas and correlates with resistance to cisplatin and clinical outcome. Clin Cancer Res.

[28] Wada M, Toh S, Taniguchi K (1998). Mutations in the canalicular multispecific organic anion transporter (cMOAT) gene, a novel ABC transporter, in patients with hyperbilirubinemia II/Dubin-Johnson syndrome. Hum Mol Genet.

[29] Materna V, Stege A, Surowiak P (2006). RNA interference-triggered reversal of ABCC2-dependent cisplatin resistance in human cancer cells. Biochem Biophys Res Commun.

[30] Kowalski P, Surowiak P, Lage H (2005). Reversal of drug-resistant phenotypes by an autocatalytic multitarget multiribozyme directed against the transcripts of the ABC transporters MDR1/P-gp, MRP2, and BCRP. Mol Ther.

[31] Remmele W, Stegner HE (1987). Recommendation for uniform definition of an immunoreactive score (IRS) for immunohistochemical estrogen receptor detection (ER-ICA) in breast cancer tissue. Pathologe.

[32] Yamasaki M, Makino T, Masuzawa T (2011). Role of multidrug resistance protein 2 (MRP2) in chemoresistance and clinical outcome in oesophageal squamous cell carcinoma. Br J Cancer.

[33] Ruggeri RM, Sciacchitano S, Vitarelli E (2006). Immunoexpression of multidrug-resistance protein 2 and cyclooxygenase 2 in medullary thyroid carcinomas. Arch Pathol Lab Med.

[34] Noma B, Sasaki T, Fujimoto Y (2008). Expression of multidrug resistance-associated protein 2 is involved in chemotherapy resistance in human pancreatic cancer. Int J Oncol.

[35] van den Broek GB, Wildeman M, Rasch CR (2009). Molecular markers predict outcome in squamous cell carcinoma of the head and neck after concomitant cisplatin-based chemoradiation. Int J Cancer.

[36] Kim YH, Ishii G, Goto K (2009). Expression of breast cancer resistance protein is associated with a poor clinical outcome in patients with small-cell lung cancer. Lung Cancer.

[37] Ushijima R, Takayama K, Izumi M (2007). Immunohistochemical expression of MRP2 and clinical resistance to platinum-based chemotherapy in small cell lung cancer. Anticancer Res.

[38] Korita PV, Wakai T, Shirai Y (2010). Multidrug resistance-associated protein 2 determines the efficacy of cisplatin in patients with hepatocellular carcinoma. Oncol Rep.

[39] Rau S, Autschbach F, Riedel HD (2008). Expression of the multidrug resistance proteins MRP2 and MRP3 in human cholangiocellular carcinomas. Eur J Clin Invest.

[40] Sandusky GE, Mintze KS, Pratt SE (2002). Expression of multidrug resistance-associated protein 2 (MRP2) in normal human tissues and carcinomas using tissue microarrays. Histopathology.

[41] Chen H, Hao J, Wang L (2009). Coexpression of invasive markers (uPA, CD44) and multiple drug-resistance proteins (MDRI, MRP2) is correlated with epithelial ovarian cancer progression. Br J Cancer.

[42] Ohishi Y, Oda Y, Uchiumi T (2002). ATP-binding cassette superfamily transporter gene expression in human primary ovarian carcinoma. Clin Cancer Res.

[43] Materna V, Pleger J, Hoffmann U (2004). RNA expression of MDR1/P-glycoprotein, DNA-topoisomerase I, and MRP2 in ovarian carcinoma patients: correlation with chemotherapeutic response. Gynecol Oncol.

[44] Guminski AD, Balleine RL, Chiew YE (2006). MRP2 (ABCC2) and cisplatin sensitivity in hepatocytes and human ovarian carcinoma. Gynecol Oncol.

[45] Ma JJ, Chen BL, Xin XY (2009). Inhibition of multi-drug resistance of ovarian carcinoma by small interfering RNA targeting to MRP2 gene. Arch Gynecol Obstet.

